# Short-term survivors with brain metastases have modest benefits from focal and systemic therapies and remain frequent despite improving treatment landscape

**DOI:** 10.1016/j.ctro.2025.100919

**Published:** 2025-01-10

**Authors:** M. Czogalla, J. Stöhr, N. Gleim, K. Papsdorf, S. Klagges, P. Hambsch, T. Kuhnt, F. Nägler, A. Barrantes-Freer, J. Wach, N.H. Nicolay, C. Seidel

**Affiliations:** a Department of Radiation Oncology, University of Leipzig Medical Center, Stephanstraße 9a, 04103 Leipzig, Germany; b Clinical Cancer Registry Leipzig, Philipp-Rosenthal-Straße 27b, 04103 Leipzig, Germany; c Department of Neuropathology,University of Leipzig Medical Center, Liebigstraße 26, 04103 Leipzig, Germany; d Department of Neurosurgery, University of Leipzig Medical Center, Liebigstraße 20, 04103 Leipzig, Germany; e Comprehensive Cancer Center Central Germany, Partner Site Leipzig, Liebigstraße 22, 04103 Leipzig, Germany

**Keywords:** Short-term survivors, Brain metastases, Radiotherapy, Systemic therapy

## Abstract

**Purpose:**

Therapeutic options for patients with brain metastases (BM) increase. While these lead to considerable survival effects in subgroups, there is limited knowledge about characteristics, prognosticators and treatment effects in patients with BM and short survival.

**Methods:**

Patients with a survival time of ≤ 6 months (short-term survivors, STS), diagnosed with BM between 2009–2021 at a large tertiary cancer center were analysed. Clinical and treatment characteristics, pathological data and causes of death were documented. Descriptive statistics, treatment-specific univariate Kaplan-Meier estimator analyses and multivariate Cox regression were performed.

**Results:**

Among 1248 patients with BM, 480 (38 %) were STS. 256 STS with detailed clinical records were included in this analysis. In univariate and multivariate analysis, Karnofsky Performance Status (KPS) (p < 0.001) and number of BM (p = 0.004) were prognostic. In 75 % of patients, the ds-GPA score predicted short-term survival. Use of resection with focal radiotherapy (p < 0.001) and systemic treatment (p < 0.001) appeared prognostically favourable compared to whole brain radiotherapy (WBRT) alone. However, survival benefits were very modest, with a median gain of 6 weeks following resection and focal radiotherapy compared to whole-brain radiotherapy, and 3 weeks from systemic treatment. Systemic tumor progression was documented as the cause of death in the majority of patients. Over the examined time period, the ratio between STS and other patients remained without significant change.

**Conclusion:**

Within STS, KPS and number of BM are of prognostic relevance. There is benefit from local and systemic therapy to a limited extent. Shared and carefully discussed individual therapy decisions are necessary.

## Highlights

• 256 patients with brain metastases and survival of less then 6 months (short-term survivors, STS) were characterized.
• KPS and BM number were prognostic, ds-GPA was able to predict STS, systemic progression was most frequent cause of death.
• Better outcome was associated with local and systemic treatment but survival benefits were only within some weeks.
• The proportion of STS among all patients remained unchanged between 2009–2019 despite improving treatment landscape.

## Introduction

During the course of their disease, 10–30 % of patients with cancer develop brain metastases (BM) and often have a poor prognosis [1], [2], [3], [4]. Over the past decades, the incidence of BM has increased, attributable to advancements in imaging techniques and prolonged survival times of patients with malignant disease, among other factors [5], [6], [7], [8], [9], [10], [11]. The medical and scientific perspectives on the disease are focused on extending life expectancy through new treatments, ultimately aiming for long-term survival. On the contrary, less is known about short-term surviving patients. Whether established prognostic factors like age, the number of BM and Karnofsky Performance Status (KPS), and prognostic scores like the disease-specific Graded Prognostic Assessment (ds-GPA) score keep their relevance in STS is unknown [1], [2], [12], [13]. Furthermore, the extent of survival benefits of local and systemic treatments, which have been shown to prolong survival in clinical trials of selected patients, are not known in patients who inherently have a very poor prognosis [7], [8], [14]. Patients with very short survival may represent a fully therapy-refractory subpopulation due to aggressiveness of disease or resistance to systemic treatment. This analysis aimed to examine all the aforementioned factors in patients with a survival of less than 6 months. Further, we aimed to analyse if the proportion of STS has decreased over the last decade with the advent of technical and pharmacological advances.

## Materials and methods

Patients that were treated in the Department of Radiation Oncology at the University of Leipzig Medical Center, Germany between the years of 2009 and 2021 for BM were identified and included. Patients ≥18 years with a maximum survival of 180 days after diagnosis of BM were eligible for this study. Survival time was defined from the day of the diagnosis of the BM until the date of death as per data of the clinical cancer registry.

For inclusion, the following variables were recorded: type of primary tumor, patient age at diagnosis of primary tumor and at diagnosis of BM, gender, KPS, number of BM, ds-GPA score and estimated survival time, actual survival time, presence of other distant metastases at diagnosis of BM, number of BM, first local therapy, systemic treatments, co-morbidities (diabetes mellitus), proliferation marker Ki-67 in patients who underwent surgery for BM and, if possible, detailed cause of death. The ds-GPA score was tabulated according to Sperduto et al. [12] for the following primary tumor entities: lung cancer (NSCLC and SCLC), breast cancer, melanoma, renal cell carcinoma and gastrointestinal cancer. For other tumor entities ds-GPA was not applicable.

Survival times were analysed with the Kaplan-Meier estimator. For univariate analyses the log-rank test was used, multivariate analysis was performed with Cox regression. The level of significance was set to α = 0.05.

To analyse the frequency of different primary tumors within different survival times a comparative subgroup analysis of patients with a survival of up to one month, a survival between 3–6 months and a group of patients that survived ≥3 years (n = 61) that was also a part of the entire cohort of patients and that we published on previously, was performed [15].

Data analysis was performed with Microsoft Excel 2024 (Version 16.81 (24011420)) and IBM SPSS Statistics (Version 29.0.2.0.).

## Results

### Patient characteristics

1248 patients with BM were recorded, of whom 480 (38 %) survived for less than 6 months. In 256/480 patients, detailed clinical information was available, of these, 40.6 % (104/256) of patients were female and 59.4 % (152/256) were male. The median age at primary tumor diagnosis was 63 years (range: 21–88). The median age at diagnosis of BM was 64 years (range: 29–90). More than half of patients were between 61–80 years old (141/256; 55.1 %). Regarding potentially relevant comorbidities, 16.8 % (43/256) of patients had been diagnosed with type II diabetes mellitus. Median KPS was 70 % (range: 30–100 %). Most frequent primary tumors were lung cancer (128/256, 50 %), melanoma (40/256, 15.6 %) and breast cancer (25/256, 9.8 %).

Main clinical and treatment characteristics incl. Ki-67 % of resected BM are displayed in Table 1.

**Table 1:** Patient characteristics including patient age, Karnofsky Performance Status (KPS), primary tumors (NFS = not further specified), number of brain metastases (BM), Ki-67 % of resected BM and treatment characteristics including systemic and local therapy. Local therapy was divided into resection, whole-brain radiotherapy (WBRT), stereotactic radiotherapy (SRT) and palliative focal radiotherapy (RT).

		**n**	**%**
Age at diagnosis of BM	18–40	7	2.7
	41–60	87	34.0
	61–80	141	55.1
	81–100	21	8.2
Grouped KPS (%)	0–30	2	0.8
	31–60	78	31.3
	61–90	157	63.1
	91–100	12	4.8
Primary tumors	NSCLC	86	33.6
	SCLC	14	5.5
	Lung cancer (NFS)	28	10.9
	Melanoma	40	15.6
	Breast cancer	25	9.8
	Colorectal cancer	10	3.9
	Renal cell carcinoma	9	3.5
	Prostate cancer	4	1.6
	Urothelial carcinoma	3	1.2
	Thyroid cancer	2	0.8
	Cervical carcinoma	2	0.8
	Other	23	9.0
	Unknown	10	3.9
Number of brain metastases	1–3	117	46.4
	> 3	135	53.6
Ki-67 % of resected brain metastases	0–20	12	25.5
	21–40	21	44.7
	41–60	9	19.1
	61–80	5	10.6
	81–100	0	0.0
Local therapy	Resection + focal RT	60	23.4
	WBRT	147	57.4
	SRT	39	15.2
	RT	9	3.5
Systemic therapy for BM	Yes	72	28.1
	No	184	71.9

The ds-GPA was calculated for 193/256 patients. 74.6 % (144/193) had a predicted survival of 0–6 months, 19.2 % (37/193) had a predicted survival of 6–12 months, 6.7 % (13/193) had a predicted survival of >12 months.

All 12 patients with a predicted survivial of more than 12 months were analysed in detail concerning further potentially prognostically intracranial and extracranial conditions. Concerning intracranial conditions, three patients had documented intracranial bleedings on brain MRI, one of these had a documented cerebellar hemorrhage as a cause of death. Two other patients had documented nodular leptomeningeal disease in proximity of the brain metastases. In four patients extensive edema or midline shift was documented (Supplement Table 1). Regarding extracranial conditions, in nine patients arterial hypertension and in four of these diabetes mellitus was documented. One patient suffered from severe coronary heart disease, however this patient died from cerebellar hemorrhage. Another patient had a documented re-entry tachycardia (Supplement Table 1).

A documented cause of death was available in 71 patients (27.7 %). In 12.7 % (9/71) of the cases, intracerebral tumor progression was the cause of death. In 83.1 % (59/71) the documented cause of death was systemic tumor progression. In 4.2 % (3/71) the cause of death was stated as unclear.

**Fig. 1:**
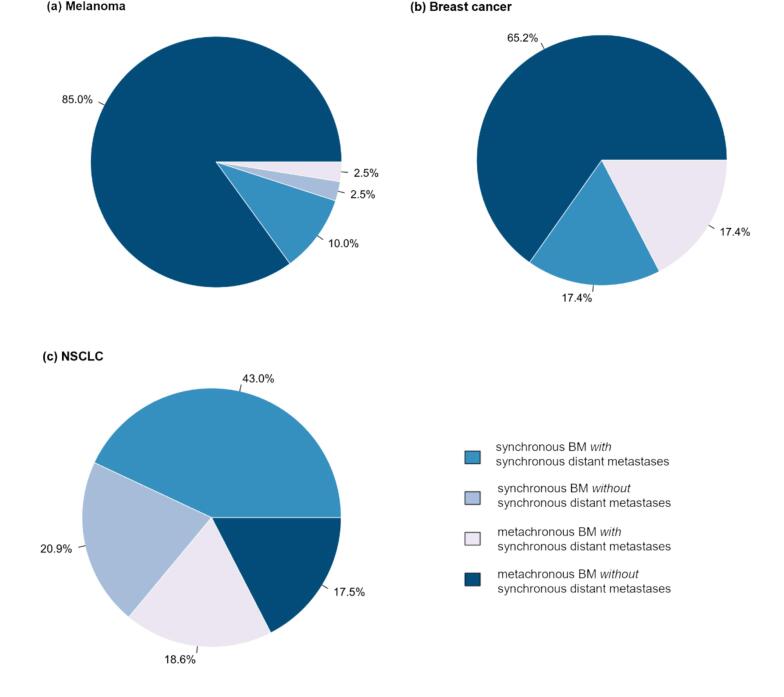
Comparison of proportions of brain metastases (BM) regarding synchronicity and distant metastases for (a) melanoma, (b) breast cancer, (c) non-small cell lung cancer (NSCLC).

In 56.6 % of cases, BM occurred metachronously and in 42.6 % synchronously. In NSCLC, the majority of BM were synchronous while the majority of patients with breast cancer and of patients with melanoma showed metachronous onset of BM (Fig. 1).

Lung cancer accounted for approximately 50 % of cases in all survival groups (1 month: 53.9 %; 3–6 months: 46.2 %; >3 years: 51.3 %). Breast cancer showed a somewhat higher representation among patients with very short or very long survival times (1 month: 14.6 %; 3–6 months: 5.8 %; >3 years: 10.3 %). Melanoma was more frequent in the group with survival between 3–6 months (1 month: 12.4 %, 3–6 months: 21.2 %; >3 years: 13.8 %) as did colorectal cancer (1 month: 1.1 %; 3–6 months: 5.8 %; >3 years: 3.4 %). Renal cell carcinoma exhibited a notable increase among long-term survivors (1 month: 2.2 %; 3–6 months: 2.9 %; >3 years: 9.9 %).

### Treatment characteristics

23.4 % (60/256) of the patients underwent a BM resection followed by irradiation of the metastasis bed, 57.4 % (147/256) received whole brain radiotherapy (WBRT) and 15.2 % (39/256) received stereotactic radiotherapy (SRT), see Table 1. A majority (61.3 %; 157/256) of patients received systemic therapy during the treatment of their disease, either for treatment of BM (28.1 %; 72/256), for a locally advanced primary tumor or distant metastases. The systemic treatment after diagnosis of BM consisted of immune checkpoint inhibitors (ICI) in 16.7 % (12/72) of the cases, 12.5 % (9/72) received tyrosine kinase inhibitors (TKI) and 70.8 % (51/72) received conventional chemotherapy.

### Survival analysis

#### KPS and number of BM are prognostic, while patient age and Ki-67 % are not

**Fig. 2:**
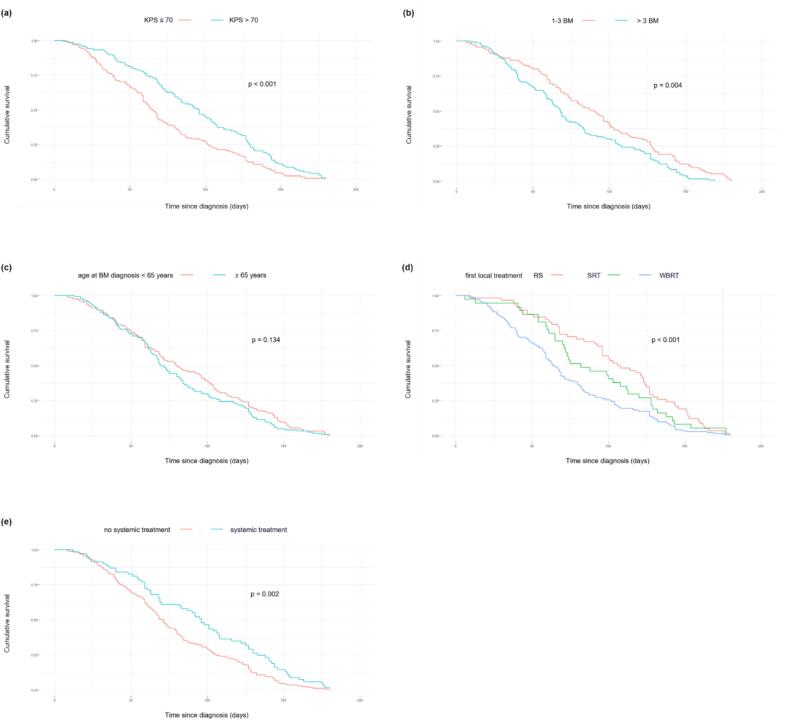
Univariate survival analysis. Associations with survival are shown in Kaplan-Meier curves for (a) Karnofsky Performance Status (KPS), (b) number of brain metastases (BM), (c) age at diagnosis, (d) type of first local treatment (resection (RS) with focal radiotherapy, whole brain radiotherapy (WBRT), stereotactic radiotherapy (SRT), (e) systemic treatment. Log-rank test p-values are shown, all associations expect age at diagnosis are statistically significant.

Patients with KPS above the median of 70 % showed significantly superior survival (median 13.9 weeks vs. 9.9 weeks, p < 0.001), see Fig. 2a. Additionally, fewer BM (1–3 vs. >3 BM, median 13.4 weeks vs. 11.4 weeks, p = 0.004) were associated with superior survival, see Fig. 2b. Age above and below the median of 64 years (median 12.0 weeks vs. 12.5 weeks, p = 0.134) did not show a significant association with survival (Fig. 2c).

In BM of resected patients, the Ki-67 % proliferation index (0–30 %: 25 patients, 31–60 %: 17 patients and 61–100 %: 5 patients) was not associated with survival (p = 0.896).

Within specific entities, higher KPS was associated with better prognosis in breast cancer (median 14.3 weeks vs. 8.1 weeks, p = 0.028) and lower BM number was associated longer survival in melanoma patients (median 16.4 weeks vs. 11.6 weeks, p = 0.002).

Other than that, no significant association of KPS, BM number and age with prognosis was discernible within NSCLC, melanoma and breast cancer patients.

#### Local treatment and systemic treatment moderately prolong survival in the entire cohort and in patients with NSCLC

Regarding treatment options, local treatment of the BM (resection followed by local radiotherapy/SRT vs. WBRT, median: 15 weeks/13 weeks vs. 11 weeks, p < 0.001) was significantly associated with longer survival in patients across all tumor entities (Fig. 2d).

Local treatment also prolonged survival within the subgroup of patients with NSCLC (p = 0.014) and melanoma (p = 0.013).

Systemic therapy during course of malignant disease (p = 0.001) and, more specifically, during the treatment of BM (p = 0.002) was associated with longer survival (Fig. 2e). Survival with and without systemic treatment after diagnosis of BM was 14 weeks vs. 11 weeks. When subdividing types of systemic treatment of BM, application of conventional chemotherapy was associated with longer survival (p = 0.012) while TKI (p = 0.144) and ICI (p = 0.440) were not. Systemic treatment after BM diagnosis was associated with better survival in NSCLC (p = 0.013, median 16 weeks vs. 9 weeks) but not in melanoma patients (p = 0.895, median 14 weeks vs. 13 weeks). In the smaller group of n = 25 breast cancer patients, no significant association of local (resection followed by local radiotherapy/SRT/WBRT, median: 18 weeks/16 weeks/9.5 weeks, p = 0.135) or systemic treatment (yes/no, median: 7.7 weeks/11.3 weeks), p = 0.116) with survival was detectable.

With regards to synchronous and metachronous BM, better survival after local treatment (resection followed by local radiotherapy/SRT/WBRT; synchronous, median: 15 weeks/11 weeks/11 weeks, p = 0.063; metachronous, median: 16 weeks/15 weeks/117 weeks, p < 0.001) was observed. In patients with synchronous BM, systemic treatment was associated with longer survival (median: 14 weeks vs. 11 weeks, p = 0.003). In patients with metachronous BM only a trend was observed (median: 14 weeks vs. 12 weeks, p = 0.116).

Median differences/gains of survival from specific treatments in different groups of patients are displayed in Table 2.

**Table 2:** Median survival difference according to patient group and treatment type. Univariate comparison and corresponding Log-rank test p-values are shown.

Patient group (n)	Comparison (n)	Median survival difference (weeks)	Survival range (weeks)	p
All patients (256)	Resection + focal RT vs. WBRT (60 vs. 147)	6	2–26	<0.001
	SRT vs. WBRT (39 vs. 147)	1	1–26	0.054
	Systemic treatment yes vs. no (72 vs. 184)	3	1–26	0.002
Patients with synchronous BM (109)	Resection + focal RT vs. WBRT (30 vs. 59)	6	2–26	0.021
	SRT vs. WBRT (17 vs. 59)	0	1–26	0.919
	Systemic treatment yes vs. no (32 vs. 77)	4	1–26	0.003
Patients with metachronous BM (145)	Resection + focal RT vs. WBRT (29 vs. 87)	7	2–26	<0.001
	SRT vs. WBRT (22 vs. 87)	6	2–26	0.14
	Systemic treatment yes vs. no (39 vs. 106)	4	2–26	0.116
NSCLC (86)	Resection + focal RT vs. WBRT (24 vs. 46)	6	2–26	0.036
	SRT vs. WBRT (14 vs. 46)	1	1–21	0.513
	Systemic treatment yes vs. no (25 vs. 61)	7	1–26	0.016
Melanoma (40)	Resection + focal RT vs. WBRT (9 vs. 22)	10	3–24	0.001
	SRT vs. WBRT (9 vs. 22)	10	3–22	0.003
	Systemic treatment yes vs. no (13 vs. 27)	1	3–24	0.904
Breast cancer (25)	Resection + focal RT vs. WBRT (2 vs. 22)	9	3–25	0.098
	SRT vs. WBRT (1 vs. 22)	7	3–25	0.235
	Systemic treatment yes vs. no (6 vs. 19)	−4	3–25	0.116

In the multivariate cox-regression analysis involving the univariate significant factors KPS, number of BM, type of local treatment and systemic treatment, KPS (HR: 0.985, p < 0.001) and number of BM (HR: 1.245, p = 0.003) were prognostically relevant. A significant association of local treatment (HR: 0.71, p < 0.001) and systemic treatment with survival (HR: 0.550, p < 0.001) persisted in this analysis.

#### Ratio of short- vs. non-short-term survivors does not change within the analysed decade

The percentage of short-term survivors with survival of less than 6 months throughout the last decade was determined (2009–2019), Fig. 3. The percentage showed no significant change over time (Chi-square test: p = 0.265).

**Fig. 3:**
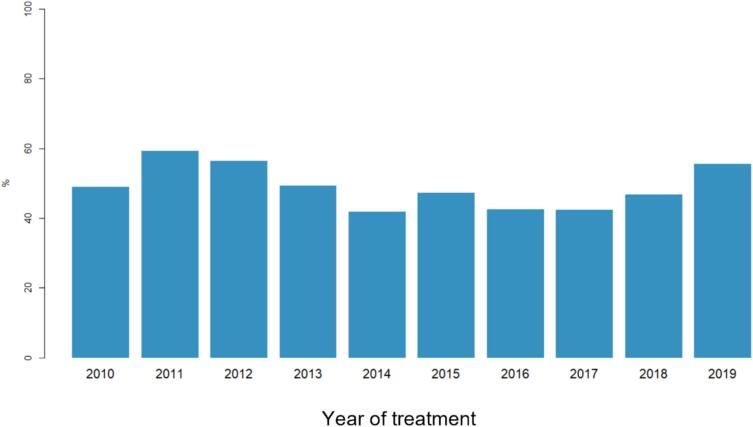
The development of the percentage of short-term survivors in our cohort over time.

## Discussion

To our knowledge, this is the first analysis of patients with BM focusing solely on patients surviving for less than 6 months. Little is known about this patient group concerning precise impact of treatments. However, detailed knowledge about prognostic factors and treatment effects is necessary to discuss different treatments or best supportive care with patients in the process of shard decision making.

According to our data, KPS and number of BM were of strong prognostic significance among STS, while age does not appear to significantly impact outcomes in this patient group. Most likely, KPS better reflects the frailty of the patient than age, and number of BM is a surrogate measure of local and general tumor aggressiveness.

In 75 % of patients, the ds-GPA score accurately predicted survival of up to six months (in 94 % up to 12 months were predicted). In this respect, our results validate the value of ds-GPA as an orientation for survival prediction, also among patients with very short survival.

Interestingly, in the minority of patients with a large discrepancy between a predicted longer survival of more than 12 months and a surviving time of less than 6 months many patients had intracranial and/or extracranial conditions that might per se contribute to a poor prognosis. For example, some patients showed intracerebral bleedings or signs of focal leptomeningeal spread on cerebral MRI reports. Further, many patients were diagnosed with arterial hypertension and diabetes mellitus. Most likely, in cases of intracranial or extractranial comorbidities with an independent mortality rate the ds-GPA cannot accurately predict prognosis. Further, the presence of leptomeningeal spread, intracerebral bleedings and diabetes mellitus are generally prognostically relevant in BM [16], [17], [18]. A further refinement of the ds-GPA with inclusion of these parameters deserves consideration in the future. Regarding the pattern of BM as synchronous or metachronous metastases, in NSCLC, patients with synchronous BM comprised most patients, while more than 80 % of patients with breast cancer and melanoma showed metachronous BM. This distribution mirrors the natural course of primary tumors, where BM occur in up to 20–25 % of NSCLC cases at initial tumor diagnosis while synchronous BM are rare in melanoma and breast cancer. As local treatments in this study, either resection with postoperative radiotherapy or sole SRT or WBRT have been applied. In the entire cohort, in subcohorts with NSCLC and melanoma, and in synchronous and metachronous settings, more intensive local treatment (i.e., resection followed by resection cavity radiotherapy or SRT) were consistently associated with better survival than WBRT alone. This effect persisted in the multivariable analysis, indicating that the association between BM number and treatment modality did not moderate this effect. In older trials, the benefit of resection of a single metastasis on overall survival has been shown [19], [20]. From our data it can be concluded that more intensive focal treatment can also improve survival in this patient cohort with an a priori poor prognosis. However, this effect was modest with a median survival gain of 6 weeks. Nevertheless, it was detectable in all entities.

A potential alternative to postoperative metastasis bed irradiation, particularly in patients with poor prognosis, is intraoperative radiotherapy which shortens in- or outpatient radiotherapy treatment period and time to systemic treatment [21].

It cannot be stated from our analysis how much WBRT has altered course of disease.

Concerning short-term survival after WBRT, the randomized non-inferiority “QUARTZ” trial did not detect a meaningful difference in survival and quality of life between patients with NSCLC unsuitable for resection or stereotactic radiotherapy, and BM with WBRT (5x4Gy) or with best supportive care only [22]. In young patients, in patients with stable extracranial tumor, in patients with a limited number of BM or a better KPS, a survival benefit has been reported [22].

With a median survival of 10 weeks after WBRT in our analysis, the limitations of this treatment should be openly discussed with patients with predicted poor prognoses, in particular in NSCLC without targetable mutation.

Improving survival in patients with predicted short-term survival requires intensive further research. Our analysis found that still today, a large proportion (38 %) of patients with BM survived less than 6 months and, somewhat surprisingly, this proportion of patients remained almost constant throughout the decade analysed. It appears that significant improvements in the treatment of BM have not resulted in a survival benefit in our cohort of BM patients with a particularly dismal prognosis. Tyrosine kinase inhibitors (TKI) and immune checkpoint inhibitors (ICI) have significantly improved prognosis in NSCLC and melanoma patients in major clinical trials and clinical practice [9], [23], [24], [25], [26], [27], [28], [29], [30]. Regarding targeted treatment of NSCLC, it is known that only a small proportion of NSCLC with ALK4 or specific EGFR alterations benefit significantly from such treatment [31], [32]. In our cohort, only 3.5 % of patients received TKI after BM diagnosis, and no survival benefit could be observed for this group. Regarding the use of ICIs, a survival benefit outside clinical trials has been observed in clinical practice, particularly in melanoma, but this benefit is less pronounced in older and frailer patients [33], [34], [35]. In our analysis, only 4.5 % of BM patients received an ICI without any apparent survival benefit. Due to this very limited number of cases, differential effects of TKI and ICI cannot be adequately estimated in our cohort. In general, systemic treatment after BM diagnosis was associated with moderately longer survival of 3 weeks in the entire patient cohort and the subgroup with synchronous BM, but no survival benefit was observed in metachronous BM. For shared decision making in patients with poor prognosis with metachronous BM, the potential lack of a significant effect of systemic treatment should be kept in mind.

Concerning cause of death, accurate data in patients with BM is very limited. Schnurman et al. performed an analysis among patients with BM and non-selected survival and found that 73.1 % died of systemic disease progression, 10.3 % died from CNS disease and 16.6 % from other causes [36]. A similar result in our cohort points to a crucial impact of general tumor aggressiveness with systemically progressing tumor in parallel to CNS-directed treatments that may control BM but not extracerebral tumor activity. The high likelihood of death from systemic failure raises the need for much better systemic treatment in patients with BM.

## Conclusion

Prognostically poor patients with BM and individual decisions for or against treatment should be more in scientific focus. The proportion of these patients remains high in recent years. KPS and the number of BM appear to be the most important prognostic factors in this patient population. Intensive local and systemic therapy may improve survival in these patients, who mostly die of systemic tumor progression. Survival gains in these negatively selected patients are illustrated in detail for the first time in this analysis but remain modest. Our analysis might aid shared and individual decision-making.

## Consent to participate

All patients included gave written consent to general scientific use of their patient-related data.

## CRediT authorship contribution statement

**M. Czogalla:** Data curation, Formal analysis, Investigation, Methodology, Writing – original draft, Writing – review & editing. **J. Stöhr:** Data curation, Investigation, Writing – review & editing. **N. Gleim:** Formal analysis, Validation, Writing – review & editing. **K. Papsdorf:** Resources, Writing – review & editing. **S. Klagges:** Formal analysis, Resources, Writing – review & editing. **P. Hambsch:** Resources, Writing – review & editing. **T. Kuhnt:** Resources, Writing – review & editing. **F. Nägler:** Resources, Writing – review & editing. **A. Barrantes-Freer:** Resources, Writing – review & editing. **J. Wach:** Resources, Writing – review & editing. **N.H. Nicolay:** Conceptualization, Resources, Supervision, Writing – review & editing. **C. Seidel:** Conceptualization, Data curation, Methodology, Supervision, Validation, Writing – original draft, Writing – review & editing.

## Funding

The authors declare that no funds, grants, or other support related to this research topic were received.
