# Supplementary material for: Short-term survivors with brain metastases have modest benefits from focal and systemic therapies and remain frequent despite improving treatment landscape
Source: Clin Transl Radiat Oncol. 2025 Jan 10;51:100919. doi: 10.1016/j.ctro.2025.100919 (PMC11772985; doi:10.1016/j.ctro.2025.100919)
Supplement: Supplementary Data 1 [file mmc1.docx]

**Supplement Table 1**

|  |  | **n (12)** | **%** |
| --- | --- | --- | --- |
| Primary tumors | Lung cancer  Breastcancer  Melanoma | 3  5  4 | 25.0%  41.7%  33.3% |
| Number BM | 1-2  3  >3 | 8  0  4 | 66.7%  33.3% |
| Synchronous distant metastasis | no  yes | 11  1 | 91.7%  8.3% |
| First Local Treatment | RS  WBRT  SRT | 4  5  3 | 33.3%  41.7%  25.0% |
